# The Prevention of Venereal Diseases

**Published:** 1916-03-11

**Authors:** 


### THE PREVENTION OF VENEREAL DISEASES.

### The Report of the Royal Commission.

In spite of the war the Royal Commission which was investigating some of the problems of the venereal diseases has continued steadily at its labours and has now presented its Report. In some ways it is perhaps unfortunate that the Report could not be held back until peace returns to our midst, for decidedly it is impracticable to expect Governmental consideration, much more Governmental action, at the present time. On the other hand, as has been pointed out, the years following a great war have in the past been distinguished for serious outbreaks of venereal disease; and if that analogy is going to hold good, the more prepared we are to cope with such a situation the better. There are many other ways in which the war will indirectly affect questions such as that of the control of venereal disease. It may be, for instance, that the regimentation of so large a proportion of the population will result in a different habit of mind among the proletariat (or rather among their political leaders) on such subjects as legislation to promote public health. Any soldier who may have seen the magnificent work of the stationary hospital in France where venereal diseases are treated (described in The Hospital of November 20, 1915, p. 161) cannot help being impressed with the advantages of compulsory notification and compulsory treatment of the most modern kind; and is far less likely to pay heed to the usual outcry of the paid agitator about the liberty of the subject and suchlike catchwords.

### The Recommendations.

The Committee have submitted a long series of recommendations with most of which there will be, we believe, a very general measure of agreement. They are opposed to any system of compulsory notification at present, but suggest that the question should be further considered in a few years' time; we take it that they are not against notification in principle, but are merely doubtful whether in the present state of public opinion it would succeed. They do, however, propose a better method of notification of deaths as part of the campaign against these diseases: the essence of which is confidential registration. The figures at present available as to the mortality attributable to venereal diseases are notoriously inexact; and one of the principal causes of this is the fact that the death certificate, stating the cause of death, has to pass through the hands of the relatives of the deceased on its way to the registrar. Hence the practitioner is obliged to conceal his real opinion for fear of causing distress to the relatives, or of incurring unjust odium himself.

### Diagnosis and Treatment.

A number of important recommendations, which do not stand the slightest chance of being carried out just yet, deal with questions of diagnosis and treatment. Laboratory methods of diagnosis and modern methods of treatment should be made "readily available for the whole community" at the public expense; and the recommendation is that three-quarters of such expenditure should come from Imperial funds, and the other quarter from local rates. We regard it as a serious blot on this part of the Report that no mention is made of contribution by the individual to the cost of his treatment. We believe the system successfully worked for some years by the L.C.C. might with advantage have been considered by the Committee: for certain of their employees the Council provide medical attendance free, except in respect to syphilis, for which they pay half only of the cost of salvarsan treatment.

The voluntary institutions will be interested to note that the Committee regard them as the most suitable agencies for providing facilities for treatment. It is recommended that local authorities preparing schemes should as their first step approach the general hospitals in their areas with a view to making arrangements for treatment. Poor-Law institutions are, however, not overlooked: facilities should be provided, says the Report, for the best modern treatment in these institutions. In prisons, too (where, as is well known, there is always a high percentage of venereal disease among the inmates), diagnosis and treatment should be made available. There are also important paragraphs dealing with the instruction of patients by various means.

Another valuable recommendation is that all advertisements of remedies for venereal diseases should be prohibited by law. These quack remedies are generally useless, and in any case they lead merely to self-diagnosis and self-treatment, and consequently encourage concealment. Again, it is proposed that the law should be amended to provide that a communication made *bona fide* by a medical practitioner to a parent, guardian, or other person directly interested in the welfare of a woman or man, and with the object of preventing or delaying a marriage with a person who is in an infectious condition from venereal disease, shall be a privileged communication; and, furthermore, that statutory recognition should be given to the principle that infectious venereal disease constitutes an incapacity for marriage. Then follow several paragraphs dealing with moral instruction of adolescents and similar educational methods of preventing venereal diseases. Although on the whole we sympathise with the general trend of these paragraphs, we are distinctly sceptical as to the value of some of them. They seem to have been drawn up without quite sufficient knowledge of human nature to achieve the results aimed at. It is noteworthy, further, that absolute silence is maintained concerning those modern methods of prophylaxis of venereal disease which have been proved to be of such high efficiency in the American Navy and elsewhere abroad. It almost seems as if the Committee, which cannot be ignorant of these important additions to scientific knowledge, had "funked" the whole subject: at any rate, the omission is both serious and regrettable.

